# Existence of multiple scales in uncertainty of numerical weather prediction

**DOI:** 10.1038/s41598-019-52157-x

**Published:** 2019-10-30

**Authors:** Hyo-Jong Song

**Affiliations:** 1 Department of Environmental Engineering and Energy, Myongji University, Yongin-si, South Korea; 2 Data Assimilation Team, Korea Institute of Atmospheric Prediction Systems, Seoul, South Korea

**Keywords:** Atmospheric dynamics, Natural hazards

## Abstract

Numerical weather prediction provides essential information of societal influence. Advances in the initial condition estimation have led to the improvement of the prediction skill. The process to produce the better initial condition (analysis) with the combination of short-range forecast and observation over the globe requires information about uncertainty of the forecast results to decide how much observation is reflected to the analysis and how far the observation information should be propagated. Forecast ensemble represents the error of the short-range forecast at the instance. The influence of observation propagating along with forecast ensemble correlation needs to be restricted by localized correlation function because of less reliability of sample correlation. So far, solitary radius of influence is usually used since there has not been an understanding about the realism of multiple scales in the forecast uncertainty. In this study, it is explicitly shown that multiple scales exist in short-range forecast error and any single-scale localization approach could not resolve this situation. A combination of Gaussian correlation functions of various scales is designed, which more weighs observation itself near the data point and makes ensemble perturbation, far from the observation position, more participate in decision of the analysis. Its outstanding performance supports the existence of multi-scale correlation in forecast uncertainty.

## Introduction

Numerical weather prediction (NWP) is a crucial technique for human health and life in modern society. Extreme weather events such as tropical cyclone^1^, heat waves^2,3^ and so on constitute continuing menaces to the society. The strength and direction of hurricane Sandy in October 2012 were predicted with 8 days lead time and the Russian heat-wave in 2010 was forecasted about two weeks ahead. The weather forecast skill has been increased by scientific and technological developments over the past decades^4^. Short- to medium-range forecast skill has been being improved by about one day per decade^5^.

Assimilating satellite data into the forecast models made remarkable progress in the NWP forecast skill increase^6^. Uncertainty in initial condition is significant enough to determine the quality of NWP^7^. Fundamental limitation of predictability comes from sensitivity of forecast error to initial conditions^8^, which is estimated at about two weeks^9^. This uncertainty of forecast is correctly represented by a group of ensemble forecast issuing from various initial conditions that have potential for being truth^10^.

If the spread of ensemble covers the true state well, it is suggested that an improved initial condition can be obtained by linear combination of ensemble perturbations with referencing observation data. In this case, the problem of estimating initial conditions is transformed into another one of determining the coefficients to control the linear combination. The ensemble data assimilation (DA) has been implemented in different flavors and the next decade is likely to be dominated by finding the most effective combination of variational and ensemble elements, which is called hybrid DA^11^.

Localization of forecast error correlation is a fundamental process to take the most advantage of introducing forecast ensemble covariance into consideration of forecast error in DA. The characteristics of the linear combination coefficient is that the more correlated the coefficient is, the stronger the correlation between variables or between time-spaces is. By allowing the co-relationship, the information propagated from neighboring observation can be retained or restricted so that the noise because of small sampling number can be discarded by decorrelation. This process is therefore called localization^12^.

**Figure 1:**
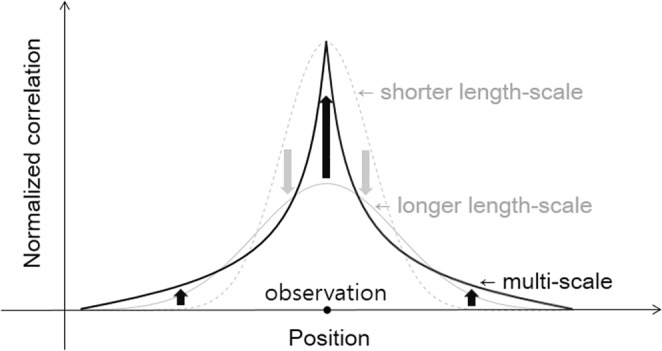
Schematic diagram of forecast error correlation function: shorter, longer, and multiple length-scale cases. These functions are representations of isotropic error correlation structure and normalized for the integration value to have one. Those are used for localization of the error correlation by element-by-element multiplication.

When it is tried for the forecast error correlation to be estimated with the Gaussian length-scale, it is explicitly observed, in this study, that any single-scale approximation to forecast error correlation is not valid. Through a forecast ensemble correlation shape, it is discovered that the correlation has multiple scales. Near observation, it shrinks steeply; its longer tail provides chances for more ensemble members to contribute to the analysis (Fig. 1). If this error correlation shape is served as a localization function, as a result, more supplies through various dynamic processes can make the analysis more reliable where the observation is rare. Even though allowing more opportunities of correction by various ensemble perturbations, the analysis result however reflects the observation in its vicinity more than the information propagated from a distant position. The multi-scale property in NWP errors is native and its consideration in DA is essential to improve the modern NWP skill.

## Results

### Characteristics of error correlation in forecast ensemble

**Figure 2:**
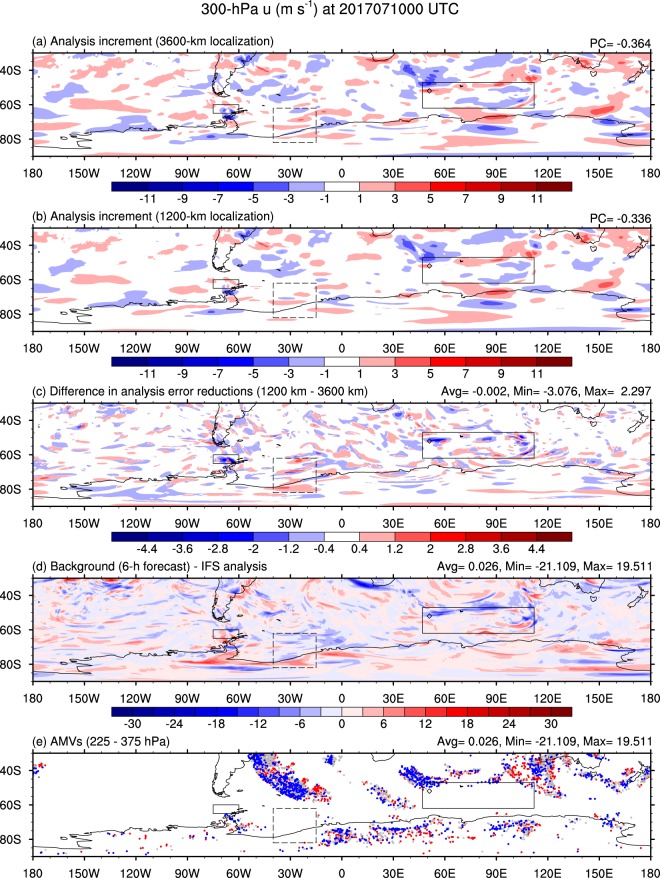
Impact of localization on hybrid data assimilation. Analysis increments of 3600-km (**a**) and 1200-km (**b**) localization approaches (PC at the upper-right corner stands for pattern correlation between background error and analysis increment) and the difference in analysis error reductions between 1200-km and 3600-km localization (**c**). The error is defined, in this study, by the difference against the Integrated Forecast System (IFS) analysis data. (**d**) Difference of background (6-h forecast) from IFS analysis and (**e**) Atmospheric Motion Vectors (AMVs) from 225 to 375 hPa (blue means zonal winds larger than +0.1 m s^−1^, red smaller than −0.1 m s^−1^, and grey in between −0.1 m s^−1^ and +0.1 m s^−1^). The solid-lined box shows longer-length scale localization takes advantage relative to shorter-length-scale one and the dashed-lined box does an opposite case. All the result are given at 0000 UTC on 10 July 2017. The diamond denotes a grid point of 52°S and 51°E.

Figure 1 shows an illustration for a type of function that is utilized for localization. Element-by-element multiplication (Schur product)^11,12^ makes propagation of the observation information asymptotically zero far from the observation. If it is too severe, the localization removes the information propagated by the correlation excessively. With comparing the analysis increments (analysis minus background) of 3600-km length-scale and 1200-km length-scale localizations (Fig. 2a,b), it is shown that the shorter length-scale makes the delivery of the observation information to the neighbor be limited. Figure 2c illustrates difference between analysis error reductions in accordance with localization length scale. A longer length-scale produces an expanded analysis increment. Where the error pattern is decoupled (dotted box in Fig. 2d), the longer analysis increment defiles the analysis (dotted box in Fig. 2c). This less-correlated error that is affected by the significant contiguous pattern of ensemble member (e.g. Supplementary Fig. 1) is corrected by localization with shortened length-scale. Where the observation is rare (Fig. 2e), the information propagated from data positions through ensemble correlation is useful occasionally (solid boxes in Fig. 2c).

**Figure 3:**
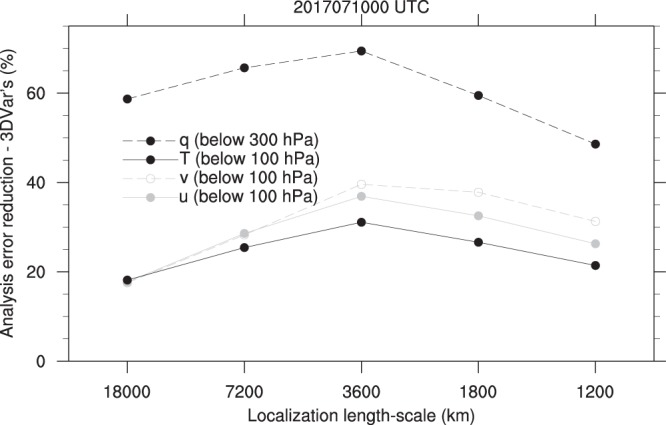
Quantitative impact of ensemble covariance localization. Difference, from 3DVar (using static forecast error covariance only), in Analysis error reduction of hybrid DA (using forecast ensemble covariance in addition to static one) with various localization length-scales, which is normalized by analysis error reduction of 3600-km length scale at 0000 UTC on 10 July 2017, for zonal (*u*) and meridional (*v*) winds (averaged over the globe below 100 hPa), temperature (*T*; averaged over the globe below 100 hPa), and specific humidity (*q*; averaged over the globe below 300 hPa). The error is defined here by root-mean-square difference (RMSD) against IFS analysis data of ECMWF.

Figure 3 illustrates the effect of forecast ensemble covariance on the analysis and the role of correlation localization. The effect of ensemble forecast covariance is defined as the difference, in the analysis error reduction (background error minus analysis error), of each hybrid DAs (using ensemble and static covariances) with different localization strategy from three-dimensional variational data assimilation (3DVar; using only static covariance). The error in this study is calculated as root-mean-square difference (RMSD) against the Integrated Forecast System (IFS) analysis from the European Centre for Medium-range Weather Forecasts (ECMWF). The difference in analysis error reduction is normalized by analysis error reduction of 3600-km localized hybrid DA. Consequently, the percentage value means how much the forecast ensemble covariance impacts on the analysis quality. The role of forecast ensemble covariance occupies 18–69% of total analysis error reduction of hybrid DA in this example. Depending on kind of variables, the significance of its impact varies: the moisture analysis is the most improved by ensemble covariance. It comes from that in the forecast error statically described in 3DVar, moisture is decoupled with other variables (wind and temperature) while winds and temperature are correlated^13^. When the ensemble forecast error covariance is used with 3600-km localization, the moisture can be corrected by the observations other than humidity itself^14^ up to 69%.

The variation of the ratio in Fig. 3 according to localization length-scale stands for how impactful the localization is in deciding the quality of resultant analysis. When the severe localization with 1200-km length-scale is applied, the impact of forecast ensemble covariance decreases down to 49% level. At least 20% of total additional skill improvement by ensemble covariance can varies depending on how we use ensemble covariance with the aid of localization. Strict localization gets rid of a possibility of using distant observations to correct moisture using multivariate correlation (solid boxes in Fig. 3). The variation in the effect of forecast ensemble covariance is the largest, 22%, for meridional wind and the smallest, 13%, for temperature; in other words, the analysis skill improvement by forecast ensemble covariance depends on localization parameter significantly.

### Existence of multi-scale in NWP forecast uncertainty

**Figure 4:**
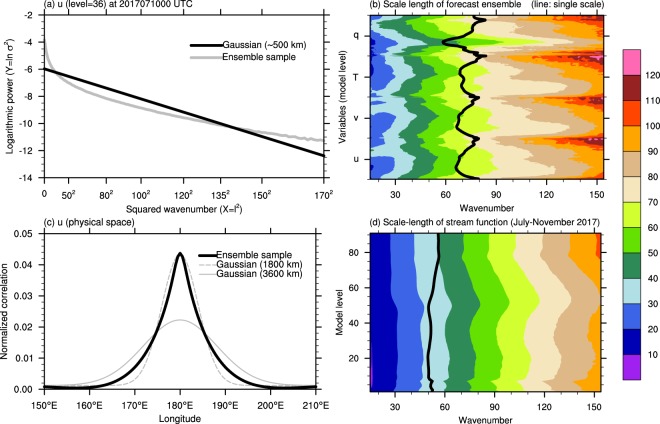
Multi-scale characteristics of forecast error in an isotropic view. (**a**) Logarithmic value of spectral power of ensemble perturbation samples (gray) and its Gaussian fitting (black) with the scale length estimated (wavenumber 67; approximately 500 km) for zonal wind (*u*) of model level 36. For the midlatitude, the wavenumber is related to a distance on the sphere by $\frac{360\,{\rm{degree}}}{{\rm{wavenumber}}}\times 100\,{\rm{km}}/{\rm{degree}}$. (**b**) Scale-lengths estimated within 30-wavenumber moving window for all variables and levels. Black line denotes single length-scale fitted to Gaussian f*u*nction. (**c**) Correlation functions of *u* in physical space from ensemble sample (black) and 1800-km (dashed gray; wavenumber 20) and 3600-km (solid gray; wavenumber 10) length-scale Gaussian function. This correlation value was normalized to have one as integration value over the longitudes. (**d**) Scale lengths according to 30-wavenumber window for 675 forecast error proxies of stream function sampled from July to November in 2017. The forecast error proxies have the same target time and different forecast lead times (36-h, 24-h, or 12-h). Black line denotes single length-scale fitted to Gaussian function.

Figure 4 illustrates that the uncertainty of NWP forecast has variable scales. Given normalized ensemble perturbations, the slope in spectral powers fitted to single-scale log-Gaussian shape informs a representative correlation length-scale: the sharp (gradual) slope stands for longer (shorter) length-scale in physical space. In Fig. 4a, a length scale differs at a certain interval of spectral band: for large scale (smaller wavenumber), the distant points are correlated each other; for smaller scale, the characteristic length-scale gets shortened. In order to find a unitary length-scale, the logarithmic spectral power is fitted by a regression line, which makes wavenumber 67 (approximately 500 km in mid-latitude), much shorter than the size of traditional synoptic pressure system that governs wind forecast uncertainty at about 300 hPa (model level 36). This existence of multiple scales and misspecified unitary representation by a short single scale are common in other variables and heights (Fig. 3b). The existence of multiple scales in forecast uncertainty is supported by the short-range (36-h, 24-h or 12-h) forecast errors of stream function sampled from seasonal duration (July to November; Fig. 3d). Therefore, it is come up that the correlation function of forecast error cannot be expressed with a single-scale Gaussian function. Micro- to macro-scales immanent in modulation of the atmospheric flow – diffusion and gravity, synoptic and planetary-scale waves – naturally induces the multi-scale property of the forecast error. Near the observation position, in physical space, it resembles 1800-km (wavenumber 20) length-scale Gaussian function (Fig. 3c); however, most of the information will be swept away far from about 10 longitude degree in 1800-km single-scale type. On the other hand, the correlation from ensemble samples is more significant than 1800-km localization function on the tail (Fig. 4c). It is therefore argued that the shape of localization function needs to be fundamentally reformulated in another way from single length-scale type.

Simply decreasing localization length-scale is not a proper approach to have the analysis skill improved (Fig. 3). A uniformly decreased scale of localization deprives hybrid DA of effectively utilizing observations especially in observation-rare areas (solid boxes in Fig. 2). On the other hand, a uniformly increasing scale of localization may have the forecast ensemble covariance exposed to sampling noise by a longer-distant analysis correction (dashed box in Fig. 2). In this regard, scale-dependent localization approach has been being developed so far^15–17^. To deal with this weakness in hybrid DA, as another approach, repeat of analysis with different scale localization has been tried^18^. The result of the Gaussian fitting of the forecast ensemble sample correlation, in this study, plainly shows the reason why a multi-scale localization approach is required. The necessity of the multi-scale localization approach is more discussed in a continuous way in Methods.

### Impact of multi-scale localization

**Figure 5:**
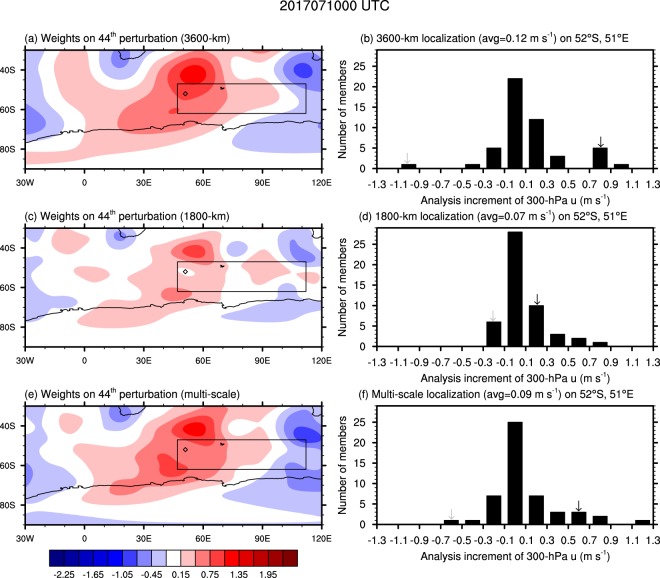
Change of the weights on ensemble perturbation depending on localization strategy. Weights on 44^th^ ensemble perturbation in 3600-km (**a**) and 1800-km (**c**) single-scale and multi-scale (**e**). The diamond denotes a grid point of 52°S and 51°E for which histograms of the 300-hPa zonal wind analysis increments are given for different localization methods (**b,d,f**). The y-axis denotes the number of ensemble members of which analysis increments on 52°S and 51°E belong to each 0.2-m s^−1^ bins. The solid-lined box shows the same area as the right one in Fig. 2. Black and gray arrows show 44^th^ and 47^th^ ensemble members, respectively.

Figure 5 shows an integrated view of the impact of multi-scale localization. In this study, multi-scale localization was implemented with gradually shortened localization length-scale from 7200 km to 1800 km (Supplementary Fig. 2). Figure 5a,c,e illustrate the weights on an ensemble perturbation, which is for the 44^th^ member (Supplementary Fig. 1), as a function of localization scale. A position denoted with diamond has no corresponding wind observation (Fig. 2e). With 3600-km (wavenumber 20) localization, the result of element-by-element multiplication of the weight and the perturbation makes an increment connecting the southwestern region to northeastern part, of the diamond position, that contains observations (Fig. 2e). With the contribution of distant observation and ensemble correlation, there is an analysis increment each ensemble member of significant magnitude (Fig. 5b). On the other hand, with a half of the length-scale (1800 km or wavenumber 20), the weights much decrease for the resultant analysis to be near zero (Fig. 5c). Since there is no observation on this point (diamond), 28 ensemble members have near-zero values (Fig. 5d) there while 22 for the 3600-km localization. It is noteworthy that the multi-scale localization makes 25 ensemble members near zero. This implies that the multi-scale localization approach weights more on the observation itself with steeply decreasing correlation according to distance, resembling the natural character of forecast error (Fig. 4c).

Although multi-scale localization makes the analysis to concentrate on the observation information itself around its vicinity (Fig. 5f), it allows a longer distant analysis increment other than 1800-km localization (Fig. 5e). While 1800-km localization decreases the analysis increment because of the distance from the observation source, the multi-scale localization retains the positive analysis increment information as +0.6 m s^−1^ (it was +0.8 m s^−1^ in 3600-km localization). The highest positive value of increment in Fig. 5f comes from longer distant observation, which implies that the multi-scale localization approach is able to put a significant weight on expanded wavy pattern (Supplementary Fig. 3). As a reversed case, for the 47^th^ member, the −1.0 m s^−1^ of the analysis increment in 3600-km localization is incorporated into around −0.7 m s^−1^ (−0.2 m s^−1^) with multi-scale (1800-km) localization (Supplementary Fig. 4). Considering the analysis increment on this observation point should have positive value to compensate the negative error (Fig. 2d), this suppression on the noise from distant observation contributes to improving the analysis skill.

**Figure 6:**
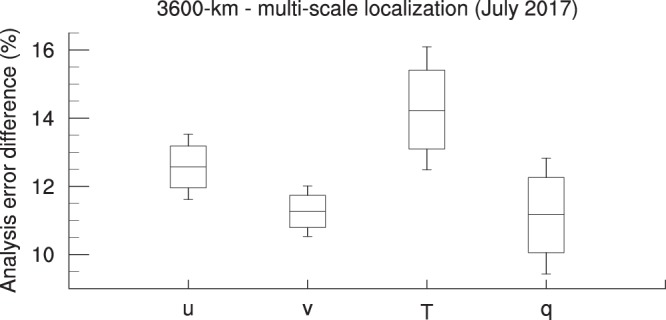
Improved performance by multi-scale localization approach. Analysis error (RMSD) difference (3600-km localization – multi-scale localization) in 1–21 July 2017, including 99% and 90% percentile area and median, is represented. The percentiles were calculated based on 100,000 bootstraps of analysis error difference percentage sampled with allowing repetition. The analysis error was normalized by the average of analysis error reduction of 3600-km localization strategy. The cycled run of DA and forecast is spun up during 23–30 June 2017.

Depending on the flowing atmospheric dynamics, propagated observation information can be useful candidate to reveal a right direction of correction^19^. With single-scale Gaussian localization function, the information on the observation position itself (with longer length-scale) or on the neighboring position (with shorter length-scale) can be underestimated. If a specific direction of analysis increment is right, more realizations will say the same directions. Therefore, the correlation function needs to be re-designed to have ‘longer tail’ for the frequency of the analysis increment realization to put higher but less and relatively even weight on longer distance. The only reference that can be used in real-time operation is observation. Therefore, the weight should be given to the observation in the vicinity of observation. If there is no observation near a model grid, the analysis increment should be given by neighboring observation so that more realizations need to be given at that point away from observation for making robust analysis result. A schematic diagram describing this principle of the multi-scale localization approach is provided in Fig. 1. As a result, Fig. 6 illustrates that multi-scale localization approach relative to 3600-km localization, which demonstrates the best analysis quality in Fig. 3, improves an analysis score up to 10–15% more. This result records 99% statistical significance based on bootstrap analysis.

## Discussion

This study investigated the structure of short-range forecast error correlation. By ensemble forecast samples and climatological forecast error samples, it is shown that the correlation cannot be dealt with a Gaussian function of a single scale-length. In physical space, the structure says that a position in the vicinity of an observation has strong correlation with the observation; however, various correlation forcings by dynamical processes (advection, stationary wave pattern and so on) must affect the position far from the observation.

Although its importance differs according to variables, the ensemble covariance localization affects the DA result significantly. Therefore, the characteristics of forecast error, the error correlation scale ranging from large to small lengths, should be considered in NWP development activities. The structure of the error correlation revealed in this study shows that a real diffusion scale is smaller than a Gaussian length-scale. As a result, the correlation value steeply drops near the observation, of which the portion lost in the near-observation is compensated on the tail of the correlation function as revealed in forecast error samples. In turn, this property provides an analysis increment with more realizations (degrees of freedom) by giving more weight on the elements in an ensemble perturbation connecting the observation to a long-distant region.

The reason why we have been using Gaussian function to describe correlations in the atmosphere is that the diffusion process makes Gaussian correlation shape. Centrifugal and Coriolis forces are critical sources to yield longer length-scale correlation so that the range that can be described by Gaussian function is shrunken and the correlation function has longer tail. When this feature is considered in designing the localization function, the advection and synoptic and stationary waves, through which observation information can be delivered, can be taken into account for obtaining the best quality of initial conditions for the weather forecasts.

## Methods

### Numerical model and data assimilation systems used

In this study, Korean Integrated Model (KIM)^20^ is used to provide 6-h forecast that is a background for data assimilation which is conducted with ‘HybDA’ of which algorithms consist of three-dimensional variational data assimilation (3DVar)^13^ and hybrid four-dimensional ensemble-variational data assimilation (hybrid-4DEnVar)^21,22^. KIM is a global NWP system, including non-hydrostatic dynamical core, based on a spectral element with cubed-sphere grid. For the details about cubed-sphere grid configuration for KIM and HybDA, Fig. 1 and A1 in Song *et al*.^23^ can be referred. In this work, the horizontal resolution of the model deterministic forecast is 25 km and ensemble forecast and DA are conducted with 50-km resolution. The 91 vertical levels are of pressure-based hybrid-sigma type^24^ with the 0.01-hPa lid.

### Hybrid data assimilation (DA)

DA a technical process that makes a result of combination of short-range forecast (called background) and observation. If the background is more reliable than observation, the information from observation should be less weighed. If the case is opposite, the background is more stuck to as it is. The reliability of background is represented by the background error covariance (BEC; **B**). The covariance is made of variance and correlation: variance means the extent of uncertainty and correlation signifies how much the uncertainty is related to neighbors and the other variables. The BEC of three-dimensional variational data assimilation (3DVar; **B**_static_) is sampled from the difference of short-range forecasts at the same target time but with different forecast lead times (e.g. 48-h–24-h) during cycled run of seasonal time scale. Since the static BEC is hard to represent ‘the Error of the Day,’ short-range ensemble forecast is utilized to do that in hybid DA as a form of the ensemble forecast covariance made of ensemble perturbations, **x**_*j*_, where *j* denotes an ensemble member. The resultant analysis increment, $\delta {{\bf{x}}}_{{\rm{hybrid}}}^{{\rm{a}}}$, of the hybrid DA is written as follows:

1 $$\delta {{\bf{x}}}_{{\rm{hybrid}}}^{{\rm{a}}}={\beta }_{{\rm{static}}}\sqrt{{{\bf{B}}}_{{\rm{static}}}}\delta {{\bf{x}}}_{{\rm{static}}}+{\beta }_{{\rm{ens}}}{\sum }_{{\boldsymbol{j}}}{{\bf{w}}}_{j}^\circ {{\bf{x}}}_{j},$$

where **w**_*j*_ is an operator to localize the correlation in the ensemble BEC, which is implicitly considered in the second term in the right hand side of the Eq. (1), and be operated with element-by-element multiplication that is Schur product (°). *δ***x**_static_ is a perturbation belonging to static BEC and following the distribution of Gaussian with the covariance **B**_static_. If **w**_*j*_ is more correlated between long-distant positions, the *j*^th^ ensemble perturbation connecting both positions through advection or wavy patterns can be more weighed and vice versa. The coefficients *β*_static_ and *β*_ens_ decide relative weightings on both types of BECs and have the following relationship:

2 $${\beta }_{{\rm{static}}}^{2}+{\beta }_{{\rm{ens}}}^{2}=1.$$

As a result, the problem of hybrid DA is transformed into finding the weights (**w**_*j*_) in addition to *δ***x**_static_ to determine the best fit to observational innovation (deviation of observation from background). The analysis, **x**^a^, is written as sum of a background (a short-range forecast of NWP), **x**^b^, and the analysis increment:

3 $${{\bf{x}}}^{{\rm{a}}}={{\bf{x}}}^{{\rm{b}}}+\delta {{\bf{x}}}_{{\rm{hybrid}}}^{{\rm{a}}}$$

### Spectral gaussian function fitting to decide correlation length-scale

To measure the length-scale of correlation in ensemble perturbation, a Gaussian fitting approach is conducted. If the spectral power (σ^2^) is obtained from ensemble samples normalized to have one as its standard deviation, a Gaussian function that best fits to the spectral power can be obtained based on least-square approach. The following function is a Gaussian representation of spectral power with a scale-length parameter, *d*:

4 $${{\rm{\sigma }}}^{2}=c\,\exp (-\frac{{l}^{2}}{{d}^{2}}),$$

where *c* is a scaling constant and *l* is wavenumber. To make the function structure linear, a logarithmic function is applied to the left and right-side hands of the Eq. (4) as follows:

5 $$\mathrm{ln}\,{{\rm{\sigma }}}^{2}=\,\mathrm{ln}\,c+(-\frac{1}{{d}^{2}}){l}^{2}$$

If we set

6 $$A\equiv -\,\frac{1}{{d}^{2}},\,X\equiv {l}^{2},\,B\equiv \,\mathrm{ln}\,c,\,{\rm{and}}\,Y\equiv \,\mathrm{ln}\,{{\rm{\sigma }}}^{2},$$

The Eq. (5) is changed into a linear relationship as follows:

7 $$AX+B=Y$$

Regression analysis for (*X*, *Y*) to obtain a regression slope *A* and an y-intercept *B*,

8 $$d=\sqrt{-1/A}\,{\rm{and}}\,c=\exp \,B,$$

Here, $d$ is the scale length of the correlation represented in spectral space, which was retrieved from the slope of the logarithmic power according to squared wavenumber as shown in Fig. 4a. For the midlatitude, the wavenumber is related to a distance on the sphere by

9 $${\rm{A}}\,{\rm{localization}}\,{\rm{length}} \mbox{-} {\rm{scale}}\,{\rm{in}}\,{\rm{physical}}\,{\rm{space}} \sim \frac{360\,{\rm{degree}}}{{\rm{wavenumber}}}\times 100\,{\rm{km}}/{\rm{degree}}$$

### Necessity of multi-scale localization approach

If the short-range forecast error involves multiple scales as suggested in this study and the shape of multi-scale error-correlation can be represented in combination of Gaussian expressions in physical space such as

10 $${G}_{mt}(x)=\sum _{k}\,{w}_{k}\,\exp (-\frac{{x}^{2}}{2{L}_{k}^{2}})$$

where w_k_ is a contribution of the *k*^th^ error correlation function $(\sum _{k}{w}_{k}=1)$ and *L*_*k*_ is a physical-spaced correlation length-scale, the necessity of multi-scale localization approach is linked to the corresponding question: can the Eq. (10) be substituted with a single Gaussian function with a single-scale correlation length-scale?

To answer this question, it is tried that the Eq. (10) is concluded to the representative single-scale, *L*, Gaussian correlation function,

11 $$G(x)=\exp (-\frac{{x}^{2}}{2{L}^{2}}).$$

For this, *G*_*mt*_(*x*) is manipulated as follows:

12 $$\begin{array}{ccc}{G}_{mt}(x) & = & \sum _{k}\,{w}_{k}\,\exp (-\frac{{x}^{2}}{2{L}^{2}}\frac{{L}^{2}}{{L}_{k}^{2}})\\  & = & \sum _{k}\,{w}_{k}{(\exp (-\frac{{x}^{2}}{2{L}^{2}}))}^{{L}^{2}/{L}_{k}^{2}}\\  & = & \sum _{k}\,{w}_{k}{(G(x))}^{{L}^{2}/{L}_{k}^{2}}\\  & = & G(x)\underline{(\sum _{k}\,{w}_{k}{(G(x))}^{\frac{{L}^{2}}{{L}_{k}^{2}}-1})}\\  & = & G(x)\sum _{k}\,{w}_{k}\,\exp (-\frac{{x}^{2}}{2}\frac{1}{{L}^{2}}(\frac{{L}^{2}}{{L}_{k}^{2}}-1))\\  & = & G(x)\underline{\sum _{k}\,{w}_{k}\,\exp (-\frac{{x}^{2}}{2}(\frac{1}{{L}_{k}^{2}}-\frac{1}{{L}^{2}}))}\end{array}$$

Based on the Eq. (12), it is revealed that the representative length-scale should be identical to all the individual length-scales ($L={L}_{1}={L}_{2}=\,\ldots \,={L}_{k}$) for the underlined part on the left hand to be one irrespective of *x*. This derivation suggests that the combination of various length-scales is required to simulate the error correlation structure if multiple scales exist in the forecast errors.

### Multi-scale localization proposed

In this study, a multi-scale localization is proposed: During total cost-function minimization process, an analysis increment is produced with localization length-scales varying through 7200 km, 5400 km, 3600 km, and 1800 km (Supplementary Fig. 2). The cost function is a metric that defines the distance of a state from the background and from the observation. The minimization process consists of outer-loop for obtaining a guess of observation and inner-loop for obtaining a state decreasing the cost function defined with the observation and guess given^21^. In this context, the multi-scale localization operates for the localization length-scale to change each outer-loop. Consequently, it makes the analysis increment obtained with aid of ensemble correlation put more weight (relative to long single-scale localization) on observation information itself near the data position in addition to the significant weight on the information propagated from longer distance (relative to short single-scale localization).

### Experiment setting

The 6-h forecast and DA have been repeated in a month from 23 June 2017 to 21 July 2017. Integrated Forecast System (IFS) analysis data of ECMWF, which record the best quality of the forecast skill (https://apps.ecmwf.int/wmolcdnv/scores/mean/500_z), are used to verify and compare the result of each experiment among different localization strategies. To show the impact of localization length-scale, several types of localization is applied to the same date, 0000 UTC on 10 July 2017 past a certain spin-up period longer than 10 days of cycled run. To produce the static BEC of 3DVar, the samples having the 36-h, 24-h, and 12-h forecast lead time difference were used, which ranges from July to November 2017 making 675 samples. The number of ensemble forecast members for forecast ensemble covariance (ensemble BEC) in hybrid DA is fifty and the initialization for the ensemble forecast was done with Local Ensemble Transform Kalman Filter (LETKF)^25^. Hybrid data assimilation was conducted with hybrid four-dimensional ensemble-variational data assimilation scheme (hybrid-4DEnVar)^26^ developed at KIAPS^14^, named HybDA^21^. Kwon *et al*.^22^ can be referred to for more details of the DA setting utilized here. For the verification of the difference between single-scale localization and multi-scale localization strategies, bootstrap analysis was used, in which 100,000 bootstraps of analysis error difference are generated with repetition being allowed^14,27^.

## Supplementary information

Supplementary information

## Data Availability

The simulation data and the Integrated Forecast System (IFS) analysis data are available from the authors upon request.

## Code availability

The code is available from the authors on request.
