# Supplementary material for: Existence of multiple scales in uncertainty of numerical weather prediction
Source: Sci Rep. 2019 Oct 30;9:15672. doi: 10.1038/s41598-019-52157-x (PMC6821884; doi:10.1038/s41598-019-52157-x)
Supplement: Supplementary file 1 — Supplementary information [file 41598_2019_52157_MOESM1_ESM.docx]

**Supplementary Information**

**Existence of multiple scales in uncertainty of numerical weather prediction**

Hyo-Jong Song^1,2*^

^1^Department of Environmental Engineering and Energy, Myongji University

^2^Data Assimilation Team, Korea Institute of Atmospheric Prediction Systems

* Corresponding author: Hyo-Jong Song, Department of Environmental Engineering and Energy, Myongji University, Myongji-ro 116, Cheoin-gu, Yongin-si, Gyeonggi-do 17058, South Korea, E-mail: hjsong@mju.ac.kr.

**Supplementary Figures**


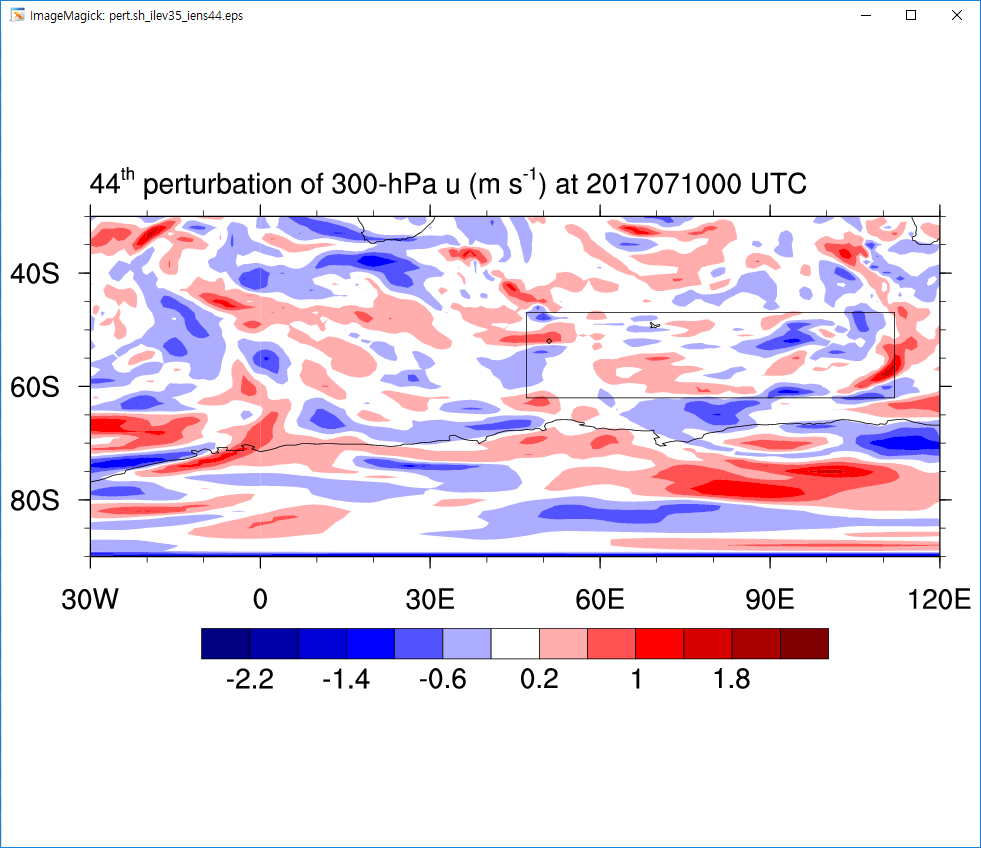


**Supplementary Figure 1. The 44^th^ ensemble perturbation of 300-hPa zonal wind at 0000 UTC on 10 July 2017.** The solid-lined box shows the same area as the right one in Fig. 2.

**Supplementary Figure 2. Localization function used for multi-scale localization.** The length-scale changes from 7200 km to 1800 km during four outer iterations of DA.


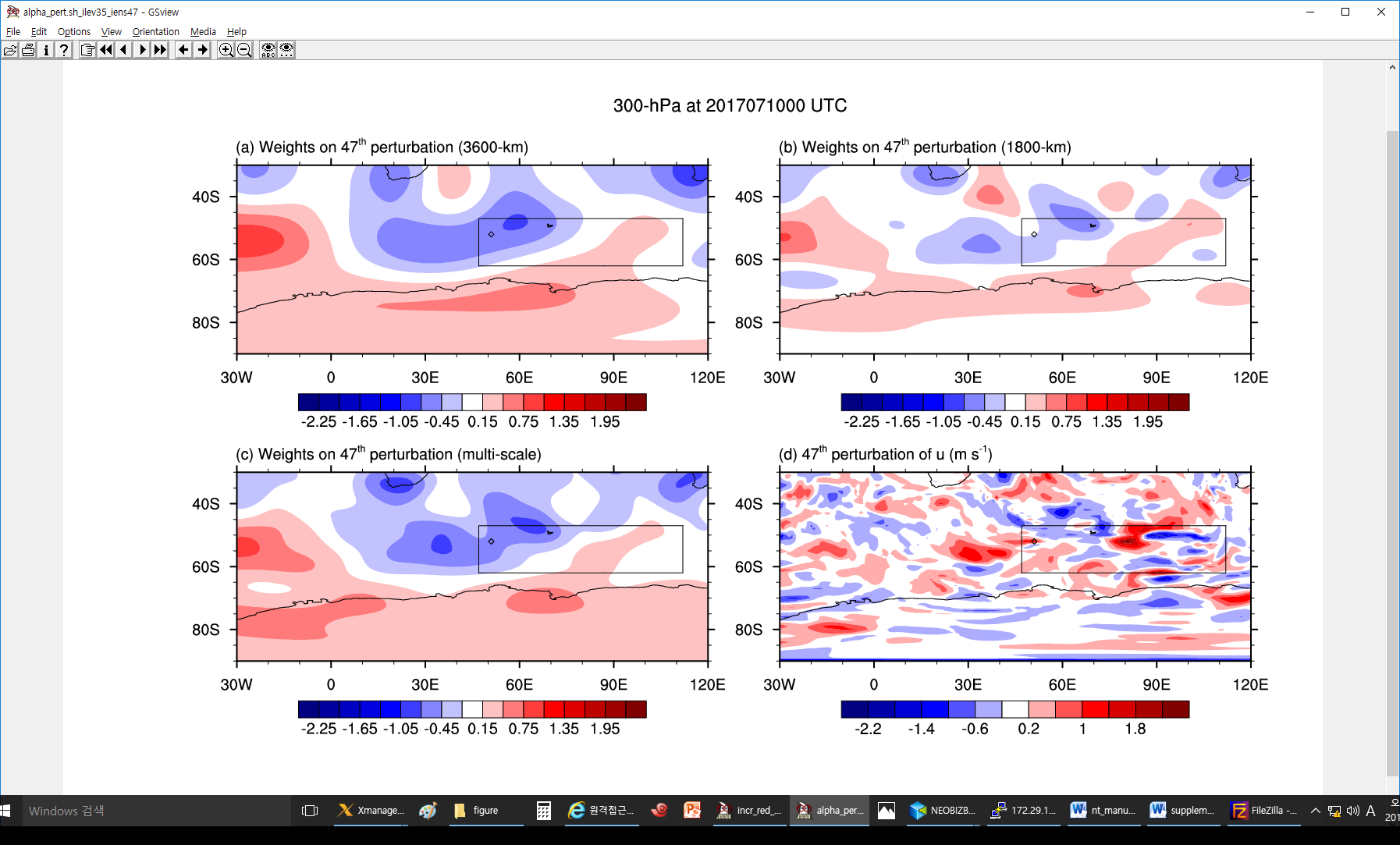


**Supplementary Figure 3. Weights (a-c) of 3600-km, 1800-km, and multi-scale localization approaches, on 42^th^ perturbation of 300-hPa u (**$\mathbf{m}\mathbf{s}^{\mathbf{-1}}$**; d).** The solid-lined box shows the same area as the right one in Fig. 2.


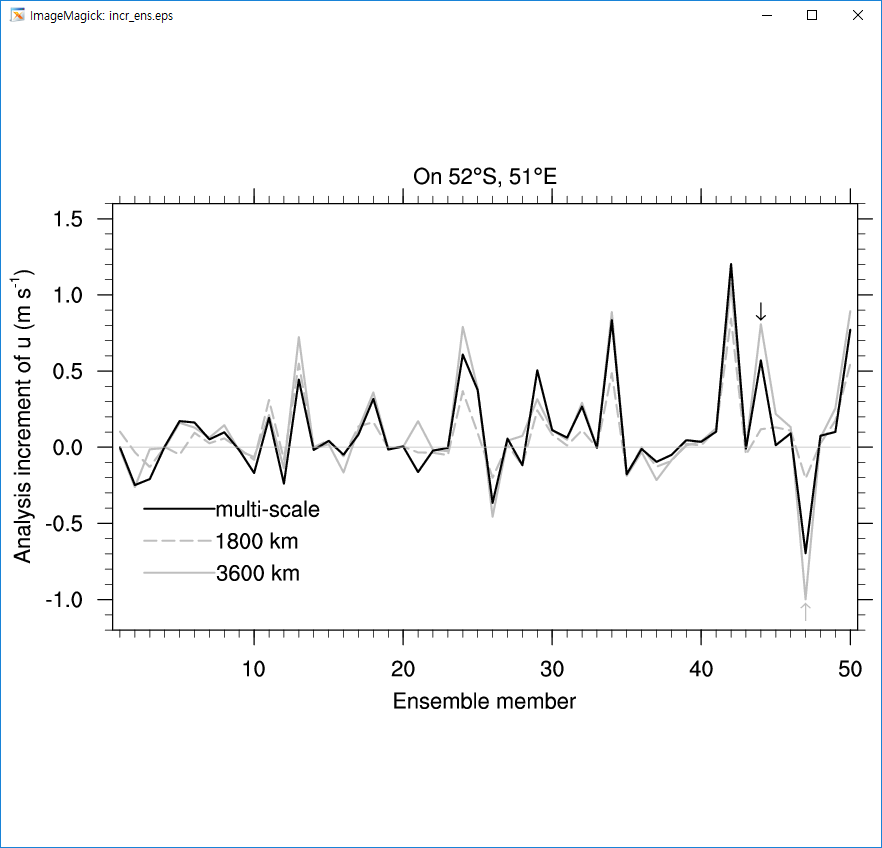


**Supplementary Figure 4.** **Actual values of 300-hPa zonal wind analysis increments for each case of Figs. 4b,d,f.** Black and gray arrows show 44^th^ and 47^th^ ensemble members, respectively.
